# Performance Enhancement of Perovskite Quantum Dot Light-Emitting Diodes via Management of Hole Injection

**DOI:** 10.3390/mi14010011

**Published:** 2022-12-21

**Authors:** Weigao Wang, Yiyang Li, Yu Duan, Mingxia Qiu, Hua An, Zhengchun Peng

**Affiliations:** 1 Key Laboratory of Optoelectronic Devices and Systems of Ministry of Education and Guangdong Province, College of Physics and Optoelectronic Engineering, Shenzhen University, Shenzhen 518060, China; 2 College of New Materials and New Energies, Shenzhen Technology University, Shenzhen 518118, China

**Keywords:** lead halide perovskites, perovskite light-emitting diodes, hole transport layers, interface modification

## Abstract

Poly(3,4-ethylenedioxythiophene) polystyrene sulfonate (PEDOT:PSS) is widely used in optoelectronic devices due to its excellent hole current conductivity and suitable work function. However, imbalanced carrier injection in the PEDOT:PSS layer impedes obtaining high-performance perovskite light-emitting diodes (PeLEDs). In this work, a novel poly[(9,9-dioctylfluorenyl-2,7-diyl)-co-(4,40-(N-(p-butylphenyl))diphenylamine)] (TFB) is applied as the hole transport layers (HTLs) to facilitate the hole injection with cascade-like energy alignment between PEDOT:PSS and methylammonium lead tribromide (MAPbBr_3_) film. Our results indicate that the introduced TFB layer did not affect the surface morphology or lead to any additional surface defects of the perovskite film. Consequently, the optimal PeLEDs with TFB HTLs show a maximum current efficiency and external quantum efficiency (EQE) of 21.26 cd A^−1^ and 6.68%, respectively. Such EQE is 2.5 times higher than that of the control devices without TFB layers. This work provides a facile and robust route to optimize the device structure and improve the performance of PeLEDs.

## 1. Introduction

Perovskites are especially promising materials for the applications of light-emitting diodes (LEDs), solar cells, and photodetectors, because of their unique properties, including tunable optical bandgap, high photoluminescence quantum efficiencies, long-range charge carrier diffusion lengths, and high mobility ambipolar carrier transport [1,2,3,4,5,6,7,8]. With these outstanding characteristics, perovskite light-emitting diodes (PeLED) have made remarkable progress in recent years, and the peak external quantum efficiency (EQE) exceeded 20% for both green and red devices [9,10,11]. Nevertheless, PeLEDs still have many problems to overcome, such as unbalanced carrier injection and transport, non-radiative recombination caused by defects at the boundary of the perovskite light-emitting layer, and poor stability.

Poly(3,4-ethylenedioxythiophene) polystyrene sulfonate (PEDOT:PSS) is one of the most popular hole transport materials in PeLEDs owing to its high transparency in the visible spectra, low-cost solution process ability, and high mechanical flexibility [12,13]. However, the perovskite films deposited on the PEDOT:PSS surface usually have poor morphology, resulting in a huge leakage current and rapid degradation of the device performance [14,15]. Furthermore, the PEDOT:PSS brings a large hole injection barrier between the work function (WF) of PEDOT:PSS (5.2 eV) and the highest occupied molecular (HOMO) of the perovskites layer, resulting in an overall charge injection imbalance between the PeLEDs and enhanced nonradiative auger recombination in the emitting layer [16,17]. There are two efficient strategies to reduce the barrier between the PEDOT:PSS layer and the perovskite layer, such as introducing an interlayer with deeper HOMO and solvent-treated PEDOT:PSS film. Lee et al. reported that a PEDOT:PSS film modified with a perfluorinated ionomer (PFI) resulted in increasing the WF and reducing the hole-injection energy barrier, and they successfully fabricated high-performance PeLEDs [18]. Yu et al. introduced an ultrathin LiF layer between PEDOT:PSS and the perovskite film, which significantly improved the surface roughness of the perovskite film and suppressed exciton quenching by the conductive PEDOT chains [19]. Some researchers have also reported the deposition of hole transport layers (HTLs) on PEDOT:PSS film to reduce the carrier injection barrier and match the valence band maximum (VBM) levels of perovskite-emitting layers, including poly[(9,9-dioctylfluorenyl-2,7-diyl)-co-(4,40-(N-(p-butylphenyl))diphenylamine)] (TFB), poly(9-vinlycarbazole) (PVK), and poly[N,N′-bis(4-butylphenyl)-N,N′-bis(phenyl)-benzidine] (Poly-TPD) [20,21]. Another effective and easier way to improve the PEDOT:PSS morphology and increase the WF is the treatment by various solvents, such as ethylene glycol (EG), [22] dimethyl sulfoxide (DMSO), [23] methanol (MeOH), [24] alkali halide (NaCl), [25] and so on. Previous studies have attempted to increase the work function of the PEDOT:PSS film to better match the energy level of the perovskite light-emitting layers (EMLs), and thereby reduce the hole injection barrier and non-radiative recombination to improve the device performance.

Herein, we fabricated high−efficiency PeLEDs based on TFB as HTLs to improve the hole injection into the EMLs with cascade-like energy alignment. This work is based on a simple synthetic approach that has been developed to produce highly luminescent crystalline methylammonium lead tribromide (MAPbBr_3_) quantum dots (QDs) as EMLs at room temperature. This approach allows for better control of the crystallization process in monodisperse nanoparticles. The introduction of the TFB layer on the PEDOT:PSS films does not influence the perovskite film morphology or the surface defects of the emitting layer and effectively reduces exciton quenching. The optimal PeLEDs with TFB HTLs show a maximum current efficiency and EQE of 21.26 cd A^−1^ and 6.68%, respectively. This EQE is 2.5 times higher than that of the control device.

## 2. Materials and Methods

Chemicals: Lead (II) bromide (PbBr_2_, 99%), methylamine (CH_3_NH_2_, 33 wt% in absolute ethanol), oleylamine (≥99%), hydrobromic acid (HBr, 48 wt% in water), and Tetrahydrofuran (≥99%) were purchased from Aladdin. Oleic acid (≥99%,, Ward Hill, MA, USA) was purchased from Alfa Aesar. Tert-butanol (analytical grade) and toluene (analytical grade) were purchased from Beijing Chemical Reagent Co., Ltd., China

Synthesis of MABr: Firstly, methylamine solution in absolute ethanol was reacted with hydrobromic acid (the molar ratio of CH_3_NH_2_ and HBr was less than 1) at 0 °C under vigorous stirring for 2 h. The precipitate was recovered using rotary evaporation under a pressure of 0.1 MPa at 60 °C for 30 min. The yellowish product was purified by diethyl ether three times and dried at 60 °C in a vacuum oven for 24 h.

Preparation of MAPbBr3 QDs: 0.08 mmol methylamine bromide (MABr) and 0.15 mmol lead bromide (PbBr_2_) were dissolved in 1 mL of anhydrous dimethylformamide, and then 500 µL of oleic acid and 20 µL oleylamine were added into the solution to form a halide perovskite precursor solution. Next, 0.5 mL of the precursor solution was dropped into 10 mL of toluene and 1 mL of tert-butanol with vigorous stirring. The product was collected after being centrifuged at 5500 rpm for 5 min, and the precipitates were redissolved into 0.3 mL tetrahydrofuran (THF) to extract the colloidal QDs. Another centrifugation at 5300 rpm for 5 min was conducted to obtain a bright green colloidal solution.

Device Fabrication: Devices with the structure of glass/ITO/PEDOT:PSS (45 nm)/TFB (40 m) /MAPbBr_3_ QDs (35 nm)/ TPBi (40 nm)/LiF (1 nm)/Al (100 nm) were fabricated. The ITO glass substrates (sheet resistance = 20 Ω sq^−1^) were cleaned by sequential ultrasonic baths in detergent, water, and isopropanol solvents for 20 min each. After drying, the substrates were treated with ultraviolet-ozone cleaner for 20 min. The PEDOT:PSS solution (Clevios AI 4083) was spin-coated onto the ITO glass substrates at 3000 rpm for 45 s and annealed at 150 °C for 15 min in air. Then, TFB solution (10 mg mL^−1^ in chlorobenzene) was spin cast on the PEDOT:PSS at 4000 rpm for 45 s, followed by baking at 120 °C for 20 min. After that, the MAPbBr_3_ QDs were deposited by spin-coating at 1500 rpm for 45 s as the emitting layer. Finally, TPBi (40 nm) and LiF/Al electrodes (1 nm/100 nm) were deposited using a thermal evaporation system through a shadow mask under a high vacuum of 5 × 10^−4^ Pa. The device active area was 4 mm^2^ where the ITO and Al electrodes overlapped.

Device characterization: The thicknesses of each functional film were measured by using a Bruker Dektak XT Stylus Profiler. The PL and TRPL were recorded by using Edinburgh Instruments FS5. The absorption spectra were characterized by a UV-violet spectrometer from the Beijing Spectrum Analysis 1901 Series. The surface morphologies were characterized via atomic force microscopy (AFM, Bruker Multimode 8). TEM images were taken by TalosF200X. A field-emission scanning electron microscope (FESEM) (Gemini SEM 300) was utilized to obtain a cross-sectional SEM image. The current density–voltage (J–V) characteristics were recorded by a programmable source meter (Keithley 2614B). The forward direction photons emitted from the devices were detected by a calibrated UDT PIN-25D silicon photodiode.

## 3. Results

**Figure 1:**
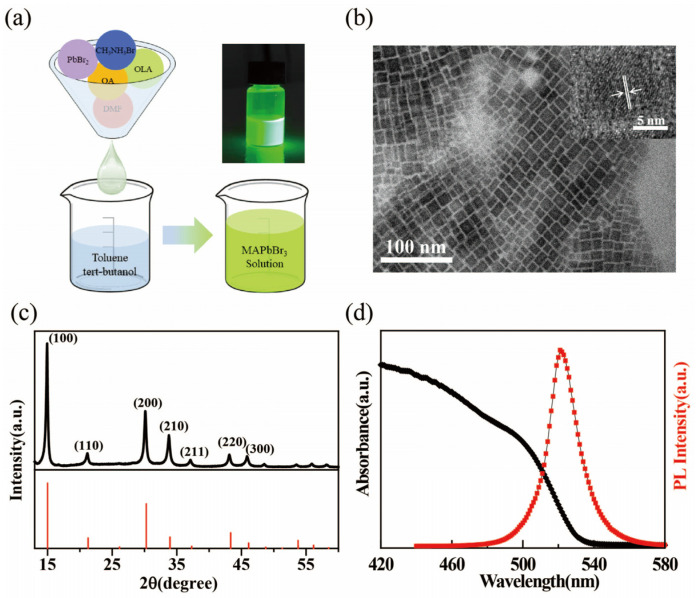
(**a**) Schematical illustration synthesis of MAPbBr_3_ QDs; inset shows a photo of MAPbBr_3_ solution under UV lamp excitation (λ = 365 nm). (**b**) TEM images and (**c**) X−ray diffraction pattern of MAPbBr_3_ QDs. (**d**) UV−vis absorption and PL emission spectra of a typical MAPbBr_3_ QDs solution.

MAPbBr_3_ QDs were synthesized by a sample ligand-assisted precipitation method at room temperature, and the synthesis process is shown in Figure 1a. A typical transmission electron microscopy (TEM) image displays the features of perovskite nanoparticle morphology (Figure 1b), where the particle size is observed to be less than 10 nm. The inserted high-resolution TEM (HRTEM) image shows that single QDs have good crystallinity. The X-ray diffraction (XRD) pattern (Figure 1c) demonstrates several characteristic peaks of MAPbBr_3_ films on 2θ of 14.86° (100), 21.1° (110), 30.1° (200), 33.7° (210), 37.1° (211), 43.09° (220), and 45.85° (300), which confirmed the formation of a stable cubic Pm3m phase. Figure 1d shows the ultraviolet (UV)−visible absorption and photoluminescence (PL) spectra of MAPbBr_3_ QDs in THF. It is observed that the MAPbBr_3_ QDs reveal a strong PL under UV irradiation. The PL spectrum shows a well−defined peak at 521 nm and a full width at half−maximum (FWHM) of 20 nm. Using a fluorescence spectrometer with an integrated sphere, we obtained a high PL quantum yield of 80–87% with MAPbBr_3_ QDs in THF solution.

**Figure 2:**
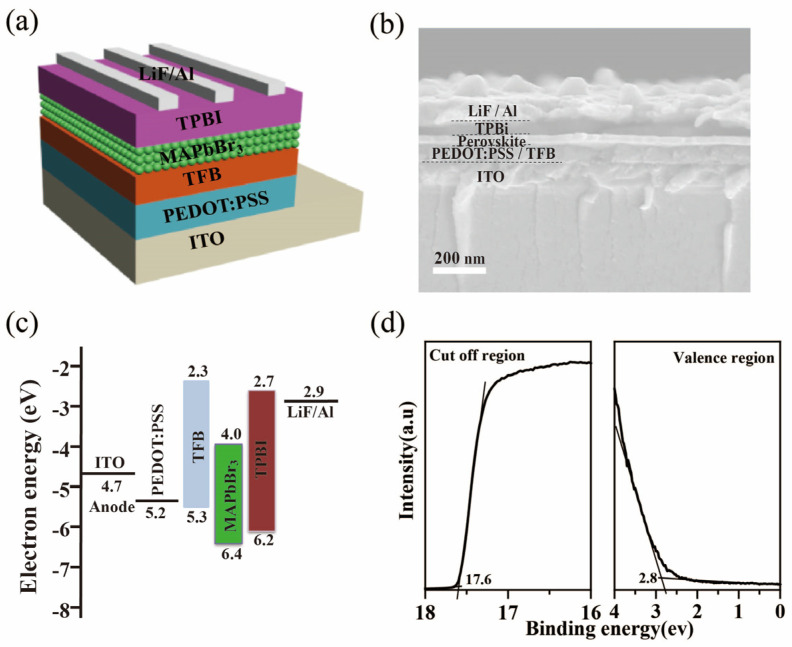
(**a**) The schematic structure and (**b**) cross−section SEM image of PeLEDs devices. (**c**) Energy level diagram for each layer of the perovskite devices. (**d**) High−binding energy secondary−electron cut−off and valence band edge regions of the MAPbBr_3_ films.

**Figure 3:**
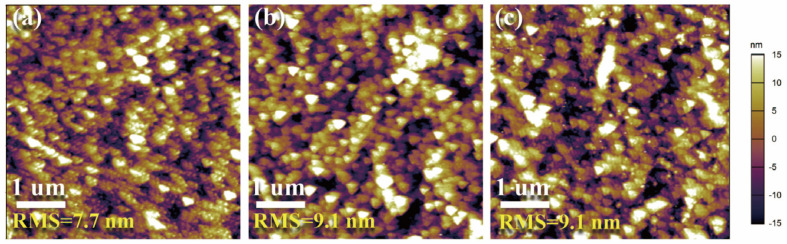
AFM images of MAPbBr_3_ films on the substrate with (**a**) ITO, (**b**) ITO/PEDOT:PSS, and (**c**) ITO/PEDOT:PSS/TFB.

In order to verify the electroluminescence performance of the MAPbBr_3_ QDs, the PeLEDs with a multilayer structure of ITO/PEDOT:PSS/TFB/MAPbBr_3_ QDs/1, 3, 5-Tris (1-phenyl-1Hbenzimidazol-2-yl) benzene (TPBi)/LiF/Al were prepared, as shown in Figure 2a. The cross-sectional scanning electron microscopy (SEM) image of the PeLED is displayed in Figure 2b, and the boundary of the multiple layers is clearly observed. Figure 2c depicts the band energy level diagram of the perovskite devices. The TFB layer as the hole transport layers (HTLs) can improve the hole injection with cascade−like energy alignment, which significantly enhances the performance of PeLEDs. To further understand the energy levels of the light-emitting layer of the device, we investigated the energy levels of the MAPbBr_3_ films by ultraviolet photon spectroscopy (UPS). The valence band maximum (VBM) can be calculated by the equation of VBM = 21.2 − E_cut-off_ + E_onset_, where E_cut-off_ is the high-binding energy cut-off and E_onset_ is the onset energy in the valence-band region [26]. According to the UPS data (Figure 2d) and the absorption spectra (Figure 1d), the VBM and the conduction band minimum (CBM) of the MAPbBr_3_ QDs are estimated to be 6.4 and 4.0 eV, respectively. Because the film morphology of the functional layer has a great influence on the performance of the devices, [27,28] atomic force microscopy (AFM) was utilized to measure the morphology of the MAPbBr_3_ film on the different substrates of ITO, ITO/PEDOT:PSS, and ITO/PEDOT:PSS/TFB. As shown in Figure 3a, the MAPbBr_3_ films on the ITO substrate show a uniform and continuous surface morphology, with a root-mean-square (RMS) roughness of 7.7 nm. The RMS of the MAPbBr_3_ films on the ITO/PEDOT:PSS/TFB is 9.1 nm, which is consistent with that on the ITO/PEDOT:PSS substrate. This indicates that the TFB functional layer has no obvious destructive effect on the surface morphology of the MAPbBr_3_ film.

**Figure 4:**
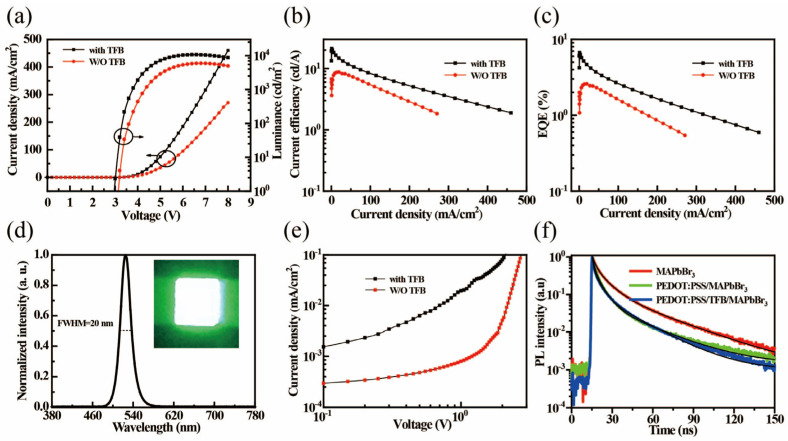
(**a**) J−V−L, (**b**) CE−J, (**c**) EQ−J characteristics of the PeLEDs with and without TFB HTLs. (**d**) Normalized EL spectra of the devices (Inset: The emission image of the optimal device driven at 6 V). (**e**) J−V characteristics for the hole−only devices with and without TFB HTLs. (**f**) TRPL decay curves of the MAPbBr_3_ QDs cast on top of ITO, PEDOT:PSS, and TFB layers, respectively.

Figure 4a shows the current density (J)–voltage (V)–luminance (L) characteristics of the PeLEDs with and without TFB HTLs. The current density of PeLEDs with a TFB layer was nearly two times higher than that of the control devices without TFB as HTLs, and their corresponding maximum luminance value ratio is also nearly two−fold. The turn−on voltage of ~2.94 V for the devices with a TFB layers is much lower than that of the devices without TFB (3.15 V) layer. This result indicates that the device with TFB as the HTLs can effectively reduce the hole injection barrier, and hence the emission efficiency of the devices. Therefore, the peaks of current efficiency (CE) and external quantum efficiency (EQE) of the PeLED device with the TFB layer were 21.26 cd A^−1^ and 6.68% (Figure 4b, c), respectively. This EQE value is 2.5 times higher than that of the control device without the TFB layer. The detailed performance data of the PeLEDs are summarized in Table 1. As seen in Figure 4d, the devices with TFB HTLs exhibit a bright green electroluminescence (EL) spectrum at a peak of 525 nm and a full width at half maximum (FWHM) of ∼20 nm. The inset of Figure 4d shows the emission image of the optimal device driven at 6 V, demonstrating a high color purity with green emission. To illustrate the effect of TFB HTLs on charge injection and transport, the hole-only devices (HOD) ITO/PEDOT:PSS/HTLs/MAPbBr_3_/4,4,4-tris(N-carbazolyl)-triphenylamine (TcTa)/N,N′-Bis(naphthalen-1-yl)-N,N′-bis(phenyl)-benzidine (NPB)/dipyrazino [2,3-f:2′,3′-h]quinoxaline-2,3,6,7,10,11-hexacarbonitrile (HATCN)/Al were fabricated. As shown in Figure 4e, the hole current density of the HOD with TFB layer is an order of magnitude higher than that of the HODs without HTLs, implying that the TFB layer is beneficial for improving the hole injection. To further investigate the effect of suppressing exciton quenching on the device performance, the time−resolved (TR) PL of the MAPbBr_3_ films on the ITO, PEDOT:PSS, and TFB layers was measured (Figure 4f). The extracted parameters from the TRPL decay curves are recorded in Table 2. The calculated average PL lifetime of MAPbBr_3_ films was slightly decreased from 19 ns to 12.5 ns, if the glass/ITO substrate was replaced by a glass/ITO/PEDOT:PSS substrate, which indicates that the excitons can be quenched by the interface defects [29,30]. However, the average PL lifetime of the MAPbBr_3_ film on the glass/ITO/PEDOT:PSS/TFB substrate is 12.7 ns, which is consistent with that on the glass/ITO/PEDOT:PSS substrate. This shows that the introduction of the TFB layer does not add additional surface defects at the interface of the perovskite film, but it does reduce the hole injection barrier and improves the efficiency of the PeLEDs.

## 4. Conclusions

In summary, we proposed a facile and simple room−temperature method to prepare high-quality MAPbBr_3_ QDs and fabricated high−performance green−emitting PeLEDs with TFB HTLs by spin−coating. The results indicate that the introduction of TFB HTLs does not affect the surface morphology of the perovskite film or add any additional surface defects at the interface of the emitting layer. By comparing the charge injection properties of HOD with and without TFB HTLs, it is noted that the TFB layer can effectively reduce the hole−injection energy barrier and improve the hole injection. The optimal PeLEDs with a TFB layer exhibit a maximum luminance, current efficiency, and EQE of 10,650 cd m^−2^, 21.26 cd A^−1^, and 6.68%, respectively. Our work provides a simple and robust method to improve the performance of PeLEDs.

## Figures and Tables

**Table 1:** Device performances of PeLEDs with and without TFB HTLs.

Device	V_on_ (V)	Lmax (cd m^−2^)	CEmax (cd A^−1^)	PE_max_ (lm W^−1^)	EQE_max_ (%)
With	2.94	10,650	21.26	19.64	6.68
W/O	3.15	5939	8.78	5.99	2.59

**Table 2:** The tri-exponential fitting parameters of time-resolved PL decay curves for MAPbBr_3_ on different substrate.

Perovskites	A1	τ1 (ns)	A2	τ2 (ns)	A3	τ3 (ns)	τ_average_ (ns)
ITO/MAPbBr_3_	0.438	2.39	0.46	8.741	0.133	32.607	19
ITO/PEDOT:PSS/ MAPbBr_3_	0.5	1.47	0.46	5.62	0.062	27.692	12.5
ITO/PEDOT:PSS/ TFB/MAPbBr_3_	0.552	1.012	0.366	5.02	0.099	22.157	12.7

## Data Availability

The data presented in this study are available on request from the corresponding author.
